# Efficacy of a self-applied online program to promote resilience and coping skills in university students in four Spanish-speaking countries: study protocol for a randomized controlled trial

**DOI:** 10.1186/s12888-020-02536-w

**Published:** 2020-04-05

**Authors:** Araceli Palma-Gómez, Rocío Herrero, Rosa Baños, Azucena García-Palacios, Claudia Castañeiras, Gabriela Lidia Fernandez, Dario Martín Llull, Lorena Cudris Torres, Libia Alvis Barranco, Leonardo Cárdenas-Gómez, Cristina Botella

**Affiliations:** 1 Universitat Jaume I, Castellón, Spain; 2 CIBER of Physiopathology of Obesity and Nutrition (CB06/03) Instituto Salud Carlos III, Madrid, Spain; 3 Universidad de Valencia, Av. Blasco Ibañez, 21, 46021 Valencia, Spain; 4 Universidad Nacional de Mar del Plata, Mar del Plata, Argentina; 5 Universidad de Buenos Aires, Buenos Aires, Argentina; 6 Universidad Popular del Cesar, Valledupar, Colombia; 7 Universidad Politécnica de Tecámac, Tecámac de Felipe Villanueva, Mexico

**Keywords:** Resilience, Online interventions, Prevention, University students, Mood disorders

## Abstract

****Background**:**

There is evidence of a high prevalence of depression and anxiety in university students. Therefore, college time is a key period where prevention of mental disorders through interventions that promote resilience and mental health can be relevant. Currently, there are interventions available, but these are insufficient for those who need them. Online interventions are tools that can facilitate global accessibility and are easy for young people to use. CORE (Cultivating Our Resilience) is a self-administered online program, based on Ryff’s psychological well-being model, to promote resilience and coping skills in university students at risk of developing symptoms of depression or anxiety. The objective is to evaluate the effectiveness of this intervention protocol in comparison with an active control condition targeting healthy lifestyle, and a waiting list control condition. The study will be conducted in four populations of Spanish-speaking university students (Spain, Argentina, Colombia, and Mexico).

****Methods**:**

The study design is a randomized controlled trial (RCT). At least 324 university students will be randomly assigned to three conditions: 1) CORE, a 6-week training program to improve resilience; 2) HLP, a 6-week training to promote a healthy lifestyle; and 3) WL, waiting list control condition. The primary outcome measure will be the Connor-Davidson resilience scale. Additionally, measures of anxiety, depression, quality of life and socio-demographic variables (age, sex, incomes, marital status, among others) will be collected. Participants will be evaluated at pre-treatment, after each module, 6 weeks after allocation, and at 3-month follow-up. Intention-to-treat and per-protocol analyses will be performed.

****Discussion**:**

The results of this study will contribute to research on Internet-administered interventions and the implementation of a protocol that includes a series of components designed to improve resilience and coping skills, increase psychological well-being, and prevent depression and anxiety disorders in Spanish-speaking university students. In addition, avenues will be opened up for new research on the effectiveness of these interventions focused on the prevention and promotion of mental health in Spanish-speaking countries.

****Trial registration**:**

Registered at ClinicalTrials.gov NCT03903978 on April 2, 2019.

## Background

Depression is the leading cause of disability worldwide [1, 2]; globally, it is estimated that 4.4% of the population suffer from a depressive disorder, and 3.6% suffer from an anxiety disorder. According to the data, this prevalence is high in the European region with 12% for depression and 15% for anxiety, but even higher in the America with 14 and 21% respectively, predominantly in women than in men [3].. This impact is associated with lower quality of life and an increased risk of developing serious physical diseases [4], loss of health and functioning, with depression being one of the contributing factors to suicide at ages 15–29 [3, 5]. In light of the magnitude, improving the health of the population is only possible if the countries involved make prevention and treatment a public health priority [6, 7].

This preventive work should focus on at-risk populations and developmental stages of the disorders. Some of the most vulnerable stages include adolescence and early adulthood [8, 9] These stages correlate with higher rates of depression and anxiety in college students, compared to the adult population, according to data from recent reviews and meta-analyses [10–13].

College life is considered a stage with high stress factors and psychological distress [14, 15]. This vulnerability is associated with various stressors, such as lifestyle changes [16, 17] or cultural challenges [18] affecting personal relationships, academic and work performance [19–22], and quality of life related to physical and mental health [23]. These factors may increase the risk of developing a mental disorder [16] or lead to self-harming behaviours and even suicide [21, 24].

The university period is, therefore, a key environment for early detection and prevention of mental illness and increased personal well-being [13, 25]. A meta-analysis indicates that the interventions with the greatest effect for depression and anxiety in university students are cognitive behavioural therapy (CBT), mindfulness-based interventions and other interventions (such as art, exercise and peer support) [26]. Some studies have found a relationship between high levels of resilience and low levels of emotional disorders such as anxiety, depression, stress, and obsessive-compulsive disorder [27]. One of the preventive approaches to increasing psychological well-being is resilience [28], which promotes protective factors of adaptation to adversity, stress, and the negative effects of exposure to risk situations [29, 30]. These protective factors will moderate and reduce the impact of negative outcomes (such as prevalence of mental disorders) and promote positive outcomes (such as mental health or psychological well-being) [29, 31].

Based on this approach, several studies have demonstrated the effectiveness of resilience interventions in reducing depressive or anxious symptoms in youth, adolescents [32–35], and university students [36]. In addition, promoting resilience has been shown to be most effective with cognitive-behavioural interventions [32] combined with mindfulness techniques [37], improving personal relationships [38], coping strategies [39], and physical exercise [40] As a meta-analysis shows, work on protective factors rather than on other variables has a greater effect on promoting resilience [41]. However, there is still no empirically validated theoretical framework describing the mode of action of resilience interventions, and so it is important to study interventions that promote resilience and prevent stress-related mental health problems [42–44].

There are other lines of research focused on the prevention of mild or moderate depressive symptoms called low-intensity psychological interventions [45–47]. One of these interventions is the promotion of Healthy Lifestyles, which shows significant clinical benefits by promoting physical health, diet, and sleep control to reduce depressive symptoms [48], and it has also been proposed for interventions in college students to prevent risk behaviours [49–51]. Although this approach regulates risk behaviours, it can be an important factor in preventing emotional disturbance, and it can be compared to resilience-based interventions.

An effective technological tool for carrying out preventive and treatment interventions in mental health is the use of the Internet [52–55]. In Europe, 79% of the population between the ages of 16 and 74 use the Internet [56], and in Latin America and the Caribbean, 55% [57]. Mainly young people are immersed in the digital world, making the Internet an effective and efficient tool [58]. The Internet is, therefore, a valuable resource for implementing empirically validated prevention programs in different regions such as Europe and Latin America, which would reduce costs by facilitating access and meeting preventive mental health objectives in at-risk populations, as established by the World Health Organization [7].

Internet-based cognitive-behavioural treatments aimed at university students have been effective in reducing psychological stress, anxiety, and depression [59–61]. A meta-analysis points to this type of intervention as a feasible method to reach this population [62], and intervention protocols to promote resilience in university students have been published [36]. Internet-based interventions [63, 64] overcome barriers such as cost, availability of services, waiting time, access, and stigma. In addition, the same effectiveness has been demonstrated as in face-to-face interventions [65–68], whether or not they are preceded by a minimum amount of therapeutic care or with other mental health professionals [69–74], Thus, it is a feasible method for the prevention of mental disorders [75–77] and the promotion of healthy behaviours [58].

Online intervention is a growing field, given the increasing rates of access to technologies to people, the reducing on cost, and the potential advantages that this way of dispense the intervention provides (e.i. the scalability, overcoming geographical barriers, increasing in flexibility on time schedule …). Studies testing this kind of approach have been mainly conducting in developed countries with high or upper-middle incomes. Lately, efforts on testing the interventions in developing countries have been done. Protocols developed or validated on Western countries started to be adapted and tested [78–81] with different methodologies [82]. The evidence on the effects in countries with middle or low income are promising [79, 83, 84], but is still scarce [85]. One potential gap is the lack of resources to properly develop, adapt, and test the interventions. In this sense, the widespread use of Spanish language provides an opportunity to test already validated interventions in other countries with the need of minor changes. Therefore, the objective of the study is to test the effectiveness of an Internet-based preventive program to promote resilience and coping skills in college students at risk of developing emotional disorders in Spanish-speaking countries: Spain, Argentina, Colombia, and Mexico, compared to a healthy lifestyle program and a waiting list control group. The hypotheses are that the CORE program will improve the resilience, coping skills, and psychological well-being of college students. In addition, CORE and active control of Healthy Lifestyle are expected to be more effective than the Waiting List control group, but with greater and statistically significant effects of CORE as an intervention that is targeting to promote resilience.

## Methods/design

### Study design

A three-armed, single blind, randomized controlled trial (RCT) with repeated measurements at three times (at baseline, 6 weeks, and 3 months follow up) will be conducted in four Spanish-speaking countries (Spain, Argentina, Colombia, and Mexico). Due to the nature of the study, the condition will be notified to participants of to which they have been assigned, but those allocated to the intervention groups will remain unaware of the type of intervention they will receive. Participants who meet the inclusion criteria will be randomized to one of the three study arms in a 1:1:1 ratio: 1) unguided internet-based resilience intervention (CORE); 2) unguided healthy lifestyle program (HLP); and 3) Waiting list control (WL). Participants allocated to the WL condition will receive access to the intervention once they have completed the evaluation period.

**Fig. 1:**
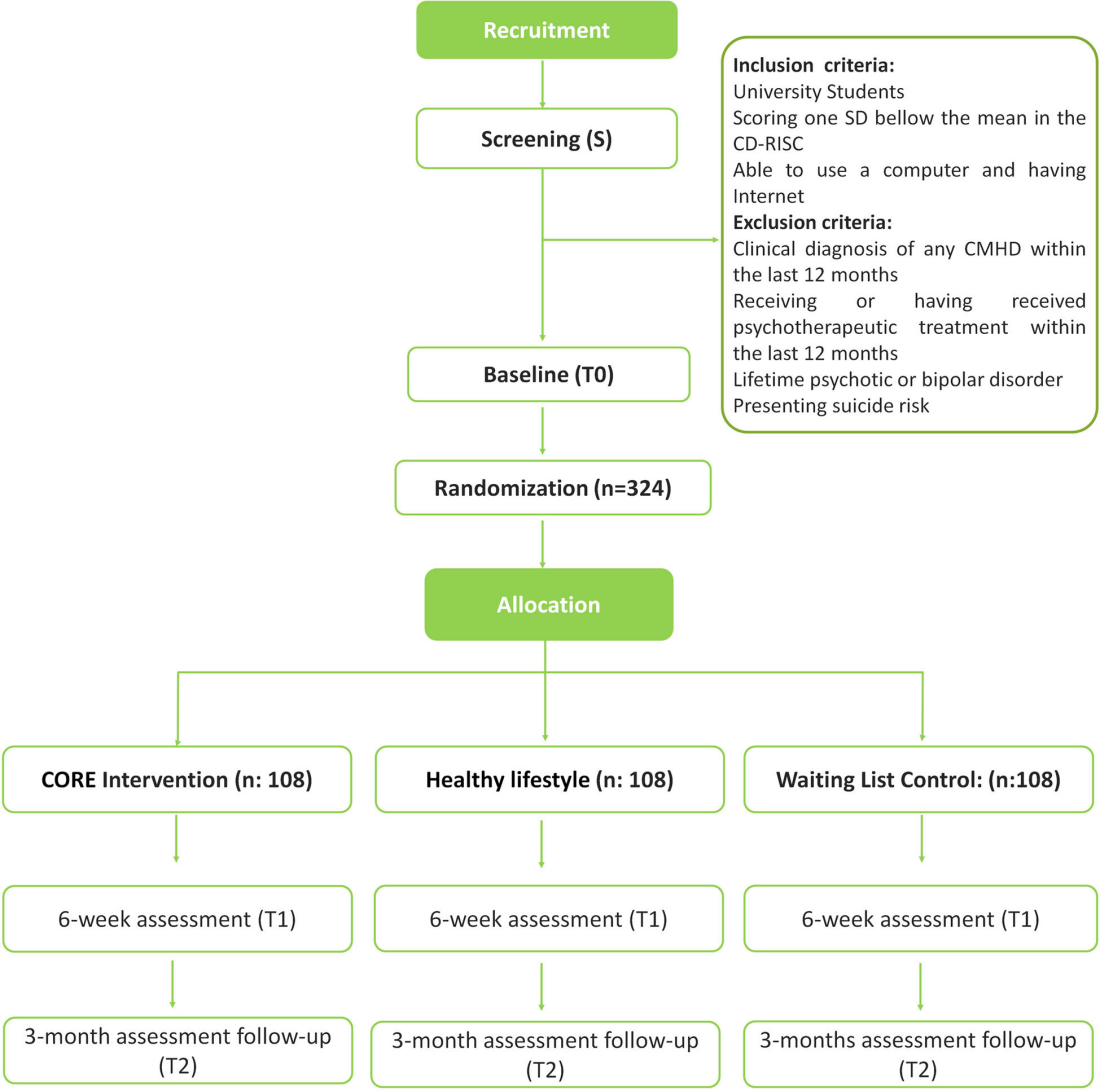
Study flowchart

The study has been approved by the Ethics Committee of University Jaume I in Spain, it was registered with the trial code NCT03903978, and it will be conducted in accordance with the CONSORT 2010 statement [86, 87], the CONSORT-EHEALTH guidelines [88], and the SPIRIT guidelines [89]. Figure 1 shows the study design.

### Sample size calculation

The primary outcome is the change in the resilience levels from baseline to post-intervention. Therefore, the Connor-Davidson Resilience Scale (CD-RISC) scores will be compared before and after the intervention in the CORE, HTP, and WL groups to obtain information about the normality of the CD-RISC scores within the target population. So far, there are no data on the effect size obtained for Internet-based interventions to promote resilience. However, a recent meta-analysis [37] compared the effectiveness of psychotherapy as an outcome for improving resilience in mental health and psychological well-being, and the authors found a moderate positive effect of resilience interventions (0.44, 95% CI 0.23 to 0.64). Considering these findings, the sample size was calculated with a significance level of 0.05, for a potency of 0.80. We take a conservative approach, we assume an effect of 0.5 (Hedges’ g), which, according to Cohen [90], can be considered a medium magnitude effect. Since our design included three experimental conditions (CORE, HLP, WL), one-way ANOVA was assumed for the statistical analyses. The total sample size calculated is 249 Estimating a potential dropout rate of 30%, the total sample size was determined to be 324, requiring 108 subjects in each condition [91].

### Study population

The participants will be college students from Spain, Argentina, Colombia, and Mexico who score low on levels of resilience (−1SD) according to the primary outcome measure.

#### Eligibility criteria

Inclusion criteria will include the following: a) College students with a standard deviation score below the sample mean on the Connor-Davidson Resilience Scale (CDRISC-25). b) Adequate knowledge to understand and read and/or speak Spanish. (c) Internet access and computer skills.

Exclusion criteria include the following, college students: a) with a CMHD record in the past 12 months; b) who are or have been undergoing psychotherapy in the past 12 months; c) with a current or past psychotic or bipolar disorder; d) at risk of suicide.

Participants who do not meet the inclusion criteria will be provided with alternative interventions that are appropriate for their needs.

#### Recruitment

Five universities in Latin America and two in Spain were contacted. These partners contributed to the management of the ethical procedures in each of the countries and to the review of the procedures and contents of the interventions. The study will be announced on the official websites and e-mail addresses of the universities: Universitat Jaume I, Castellón, Spain, https://www.uji.es/; Universitat de Valencia, Spain, http://www.uv.es/; Universidad de Buenos Aires, http://www.uba.ar/; Universidad Nacional de Mar del Plata, Argentina, http://www.mdp.edu.ar/; Universidad Autónoma del Estado de Hidalgo, Mexico, https://www.uaeh.edu.mx/; Universidad Politécnica de Tecámac, México, http://uptecamac.edomex.gob.mx/; and Universidad Popular del Cesar, Colombia, https://www.unicesar.edu.co/. It will also be communicated through public spaces such as student social networks (e.g. Facebook, Instagram, and Twitter), posters, and newspaper advertisements. Interested students will request participation by email. A link with the informed consent and the screening criteria will be sent in reply.

### Interventions

#### Intervention (CORE)

CORE is a program developed within the framework of the ICare Project (H2020 No. 634757), and good results were obtain in a previous study conducted in Spain, Germany, and Switzerland [63]. This program is a prevention protocol with therapeutic components based on empirical evidence following the Ryff wellbeing model [86, 92] and organized in six dimensions: autonomy, self-acceptance, mastery of the environment, purpose in life, positive relationships, and personal growth. The main objective is to teach coping skills and strategies to cope with stressful everyday situations in order to improve resilience, promote self-efficacy, and increase well-being. The intervention consists of six interactive modules designed for weekly sessions (see Table 1), and it includes exercises to practice the skills proposed in each module. CORE is a program designing to increase resilience following Ryff wellbeing model [86, 92], therefore along the use of the program the participant will be.

**Table 1:** CORE modules and their objectives

Module	Objective
0. Welcome	Introduction module to the program, with an explanation about the tools and the way to use CORE
1. Psychoeducation	Explanation of psychological wellbeing and the concept of resilience: - Understand the concept of psychological well-being, its most important aspects, and their relevance in life. - Understand the concept of resilience and the importance of training and cultivating it.
2. Autonomy: building my way	Enhancement of autonomy: - Develop a healthy lifestyle (by pursuing balance in several areas: activity, food, sleep). This lifestyle will allow the person to focus on his/her goals in life. - Increase psychological well-being by working on abilities potentially related to values and goals in life.
3. Mindfulness and self-compassion	Training in mindfulness, savoring, and an attitude of self-compassion: - Learn the meaning of “mindfulness”, how to develop this ability, and the benefits that its practice can bring. - Learn to distance ourselves from our thoughts and how to handle them. - Understand the importance of, recognize, capture, and enjoy the good moments. - Develop the skill of kindness and self-care, i.e., the capacity for self-compassion.
4. Overcoming obstacles	Development of coping strategies to deal with daily difficulties in life: - Be aware of the importance of facing problems properly. - Learn the Problem Solving Technique and how to apply it. - Learn the role of our thoughts in the way we feel and how to be flexible in our way of interpreting situations.
5. Connecting to others	Acknowledge the relevance of relationships and how they can be helpful in the construction of well-being: - Recognize the importance of our social relations. - Learn to care for and improve our social relations. - Learn to promote quality relationships, which can contribute to maintaining and strengthening resilience.
6. Purpose in life and personal growth	Encourage students to deal with the future with a positive attitude, taking into account what is important for each person and planning the future according to these objectives.

Users will be recommended to perform one module per week, which will be automatically activated on the platform, Participants will have a total of 6 weeks to complete the program prior the activation of post assessment, were the modules will remain active. Post assessment and 3 months follow-up will be automatically activated by the platform. Participants can leave the intervention at any time. Once the post-treatment assessment has been completed they will have free access during the next 3 months, either to enter and use the unfinished modules or for revisit them at any occasion. The system will track any access to the content. The intervention has a series of task and homework to assist the participant in the acquisition or development of the proposed skill. Tasks and homework are proposed along the intervention and their fulfilment are tracked by the system.

**Fig. 2:**
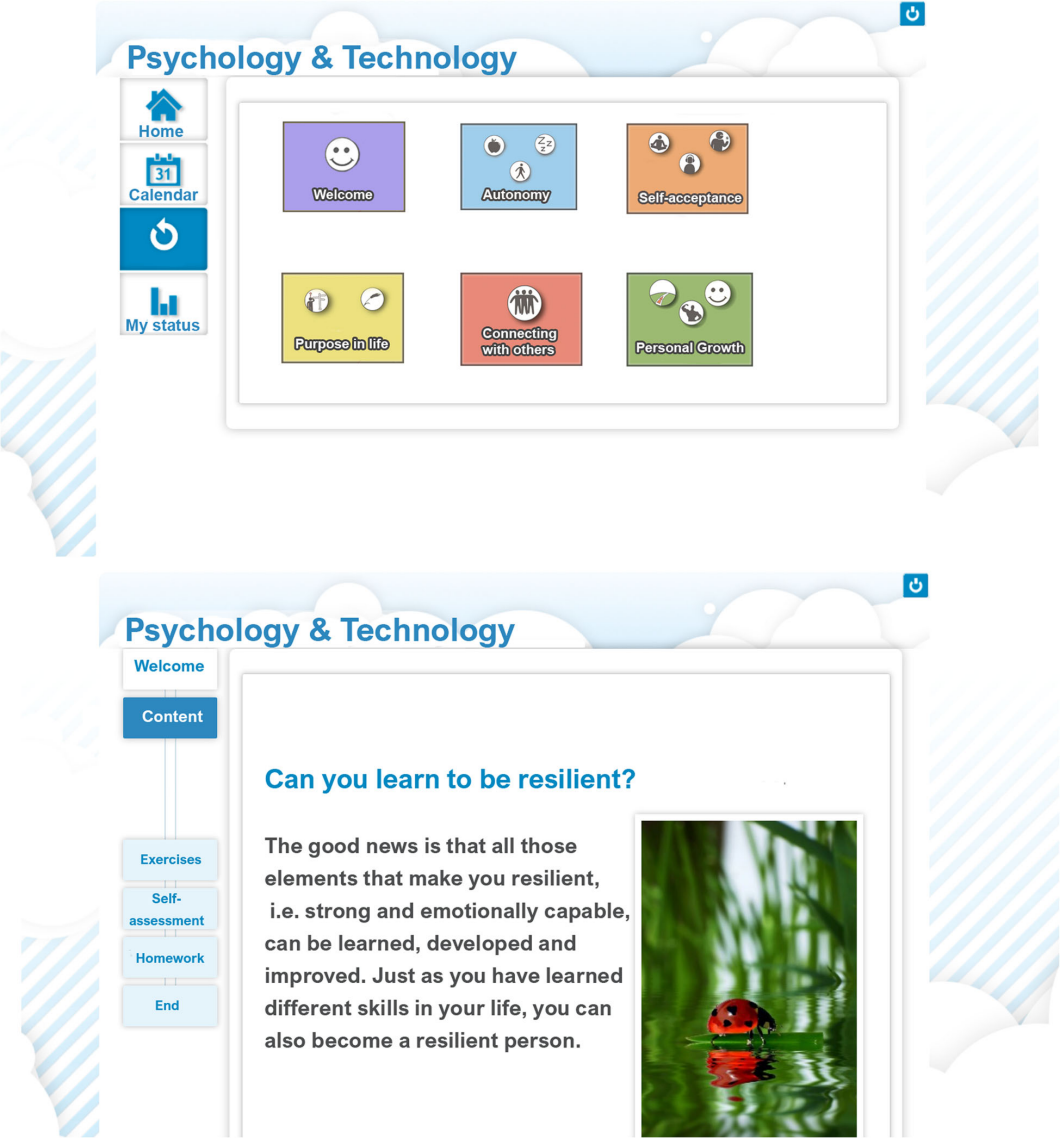
CORE training: multimedia tools within the platform: https://www.psicologiaytecnologia.com/

The program was financiered and developed within the ICare project (H2020 No. 634757). Pilot studies and focus groups with college students and health professionals were carried out for its development. It was adapted to the web format, adding multimedia material (videos, audio, and images), and verification questions (see Fig. 2). Previous data on acceptability shows good rates among users. Currently, the preliminary results on effectiveness are good. In addition to the content the participants receives weekly support messages with general information regarding the tasks participant must work during the week. These messages will be included in both active conditions (CORE, HLP), and are predesigned (see Table 2).

**Table 2:** Example of the e-mail support protocol per module for the follow-up of the participants

Module/subject	Message
1.1 Session 1 – First week reminder Subject: CORE – Module 1	Dear X, / Session 1 is available for you! / We want to invite you to take the time to do the training. The training can give you helpful ideas, especially when you are very stressed with your daily life. It will help you to live a more relaxed and aware life. / We would be happy to hear from you soon and wish you all the best. / Best wishes /Y from the CORE-Team
1.2 Session 1 – Second week reminder Subject: CORE – Module 1	Dear X, /We know that it isn’t always easy to integrate the training into your daily life, particularly when you have a lot of other commitments. Nonetheless, we want to encourage you to find some time to work on the training. / In session one, you learn what it means to be resilient, and you get useful tips that can help you to become more resilient. / We would be glad to hear back from you shortly. / Best wishes / Y from the CORE-Team
1.3 Session 1 – Third week reminder Subject: CORE – Module 1	Dear X, / Please think about this: To profit from the training, it is important, to stay on track. Therefore, it is not only important to integrate the exercises into your daily life, but also to work on your next session. We also want to encourage you to find some time to do the first session to improve your well-being actively. / If you have any questions, don’t hesitate to contact us. Best regards / Y from the CORE-Team

All modules have a common structure, starting with a scheme of the most relevant points to work during the session, followed by the content itself. At the end of the module, exercises and self-test questionnaire are presented to verify whether the explanation has been understood and provide feedback either if the answer is correct or not. In addition, at the beginning of each module, a verification test is provide to see the degree of adherence to the tasks proposed. CORE has as main goal to impact on the resilience and wellbeing of participants. To do so the intervention content of 6 interactive modules. In the first module the participant receive an explanation of psychological wellbeing and resilience and the importance of training and cultivating it. The second module is focus on the development of a healthy. The third module is aimed to train mindfulness, savouring, and an attitude of kindness and self-compassion. On the fourth module the objective is to give information on how to overcome obstacles by learning the Problem Solving Technique. On the fifth module, participants will be instructed in the relevance of relationships and how they can be helpful in the construction of well-being. Participants will be taught about the ways to promote quality relationships. In the last module participants will be encourage to have a positive attitude toward the future, focusing on what is important for each person and planning the future in accordance.

CORE will operate on the web platform: Psychology and Technology (https://www.psicologiaytecnologia.labpsitec.es/). The platform allows researchers to produce the content of the intervention and deliver it to the participants. It also ensures secure and encrypted communication between clients and the researcher. Given that the study will be conducted in different countries and the intervention is self-apply, user guidelines for researchers and end users were made. The guidelines content information about the ways to access the platform, common questions, and a support protocol for researchers. Beside the platform counts with a support system, that provides help on technical issues to users.

The content of the program and messages was reviewed by researchers from Latin American countries, and it was tested in small pilot study in each country. The content was considered adequate, and small changes were made in the wording.

#### Healthy lifestyle program (HLP)

HLP is a Healthy Lifestyle Program was developed under the framework of a national project in Spain funded by Carlos III Institute. The program has been tested and showed to be effective in reducing depressive symptoms in patients with mild to moderate depression. Its background supports its usefulness for improving the person’s lifestyle and reduce depressive symptoms. The psychoeducation components are based on the protocol of low-intensity psycho-pedagogical intervention for depression applied by ICT models [93, 94] for mild or moderate depressive symptoms in primary care [45–47] and the prevention of depressive symptoms [95], whose components have been shown to be effective [94]. HPL is a program that provides information to promote a healthy physical and mental lifestyle regarding topics related to physical activity, diet, and sleep management (see Table 3). The intervention consists of four interactive modules designed as weekly sessions, and it includes multimedia elements: videos, audios, vignettes and images.

**Table 3:** HLP healthy lifestyle and their objectives

Module	Objective
1. Beginning to change lifestyle	Learn to identify healthy behaviors and risks in order to recognize obstacles that impede adopting a healthy lifestyle. Teach hygiene and eating strategies to improve emotional health and well-being.
2. Learning to feed ourselves	Teach the importance of a balanced diet in maintaining good physical and mental health. The Mediterranean diet will be taken as an example of a balanced diet. Obstacles to maintaining a healthy diet will be analyzed to prevent any depressive symptoms.
3. Moving our body	The importance of “moving” and activating behavior will be taught through regular exercise routines to improve mood. Training will be provided to increase motivation, activity, and fitness.
4. The importance of good sleep	Strategies will be provided to understand the relationship between sleep and overall health, emphasizing the importance of good sleep in maintaining a good mood.

The whole program has a common structure for all modules. Each module starts with a scheme of the most relevant points to work during the session, followed by the content itself. At the end of the module, different exercises are proposed, and self-test questionnaire is presented to verify whether the explanation has been understood and provide feedback either if the answer is correct or not. In addition, before starting each module, a verification test is provide to see whether the participants have carried out the proposed tasks and responds by congratulating them or encouraging them to do the task. HLP has as main goal to impact on the life style of participants. Therefore the content gives information regarding the relation between a healthy life style and wellbeing, the importance of carry out regular physical activity. Advices regarding diet are provided, giving information about the Mediterranean diet, along with education on 6 healthy dietary commands. All of this will come with information and practical exercises on how to systematize food and activity-related behaviours. In addition, the importance of developing a social support network will be emphasized. People will be shown specific techniques on how to create and maintain an adequate social support network.

Participants will have a total of 6 weeks to complete the program prior the activation of post assessment. Post assessment and 3 months follow-up will be automatically activated by the platform. Participants can leave the intervention at any time. Once the post-treatment assessment has been completed they will have free access during the next 3 months, either to enter and use the unfinished modules or for revisit them at any occasion. The system will track any access to the content. The intervention content a series of task and homework to assist the participant in the acquisition or development of the proposed skill. Tasks and homework are tracked by the system.

The program was financiered and developed within the Carlos III Institute. Pilot studies and focus groups were carried out for its development. It was adapted to the web format, adding multimedia material (videos, audio, and images), and verification questions. Currently, the preliminary results on effectiveness are good. In addition to the content and in order provide the same support that in CORE condition; the participants will receive weekly support messages with general information regarding the tasks participant must work during the week.

HLP has been manualized and adapted for the web, and it will be use on the web platform Psychology and Technology. The procedures for its use will follow the same structure than in CORE condition.

The content of the program and messages was reviewed by researchers from Latin American countries, and it was tested in small pilot study in each country. The content was considered adequate, and small changes were made in the wording.

#### Waiting list (WL)

Participants assigned to the Waiting List Control condition will be evaluated and monitored prior to the beginning of the intervention, at 6 weeks, and in a follow-up at 3 months. After the last evaluation, they will receive access to the CORE intervention.

### Outcomes

All the assessments will be conducted online in each country using the Psychology and Technology platform. Table 4 provides an overview of the measures and their time of assessment.

**Table 4:** Study measures, time and type of assessment

Questionnaire	Construct	SCREENING (S)	PRE (T0)	PRE-POST MODULES	POST (8 weeks) (T1)	FU (6 months) (T2)
Socio-demographic data	Demographics	X	–	–	–	–
CD-RISC −25	Resilience (primary outcome)	X	X	–	X	X
ODSIS	Overall Depression Severity and Impairment Scale	–	X	X	X	–
Suicidal ideation	Risk of suicide	–	X	X	X	–
PHQ-9	Depression severity	X	X	–	X	X
BFI-10	Personality inventory	–	X	–	–	–
GAD-7	Anxiety symptoms	–	X	–	X	X
EAHF	Openness to the future scale	–	X		X	X
CSQ	Treatment satisfaction	–	–	–	X	–
PANAS	Positive and negative affect	–	X	–	X	X
PWBS-29	Psychological well-being	–	X	–	X	X
SCS-SF	Self-compassion	–	X	–	X	X
RPA	Response to positive affect	–	X	–	X	X
PSS-4	Perceived stress	–	X	–	X	X
OASIS	Overall Anxiety Severity and Impairment Scale	–	X	X	X	–
CEQ	Credibility and expectancy of treatment	–	X	–	–	–
WAI-TECH	Therapeutic alliance	–	–	–	X	–

### Primary outcome

#### Resilience

*Connor-Davidson’s Resilience Scale (CDRISC)* [96] assesses stress coping skills using a 25-item self-report questionnaire that uses a five-point Likert scale ranging from 0 to 4 (0 = strongly disagree, 4 = strongly agree). Scores range from 0 to 100, with higher scores reflecting greater resilience. Previous studies show that it has good internal consistency (Cronbach alpha above 0.70) [97–100].

### Secondary outcomes

#### Well-being

*The Ryff Scales of Psychological Well-Being - 29 items* (PWBS-29) [86] is an instrument for measuring the faces of psychological well-being, including the six dimensions of the Ryff model (autonomy, self-acceptance, mastery of the environment, personal growth, positive relationships with others, and purpose in life). Response scores range from 1 to 6 (1 = strongly disagree, 6 = strongly agree). This scale has been shown to have good psychometric properties [87, 88].

#### Positive and negative emotionality

*Positive and Negative Effects Program (PANAS)* [89]. PANAS evaluates two independent dimensions: positive affect (PANAS+) and negative affect (PANAS-). It consists of 20 items divided into two dimensions with 10 items each, with scores ranging from 10 to 50. It has shown good properties of validity, convergence, and divergence, and it is a brief, reliable self-report measure [101].

The Responses to Positive Affect questionnaire (RPA) [102] is a questionnaire that assesses the responses to positive affective states and consists of 17 items. Items are rated on a four-point scale, ranging from 1 (almost never) to 4 (almost always). The original measure consists of three factor-analytically derived subscales: Dampening, Self-focused positive rumination, and Emotion-focused positive rumination. Initial psychometric results with the original English version show adequate reliability, validity, and internal consistency for each scale (α = 0.76, 0.72, 0.73 for Factors I: Emotion-focus –III: Self-focus, respectively) [102]. Discriminant validity has been supported because the scale is uniquely related to risk of hypomania after controlling for other measures of impulsivity and responses to positive affect [103].

*Openness to the Future Scale (OFS)* [104] consists of 10 items with scores ranging from 1 to 5 on a Likert scale. It assesses expectations and positive affectivity towards the future, which includes five domains: (1) Illusion of control, (2) Acceptance, (3) Commitment to life and planning, (4) Positive orientation towards the future, and (5) Self-efficacy towards the future. The Openness to the Future scale shows similarly good convergent and discriminant validity across clinical and community samples. Cronbach alphas for the 10-item scale were acceptable for both the clinical (.82) and community samples (.87), with cut-off points from 37.5 to 38.

#### Depression and anxiety

*The Patient Health Questionnaire (PHQ-9)* [105] is used to examine and diagnose patients with depressive disorders. It consists of nine-items measured on a scale from 0 to 3 (0 = not at all, 3 = almost every day). Total scores range from 0 to 27. The severity cut-off points for depression are 5, 10, 15, and 20, and they represent, respectively, the thresholds for mild, moderate, moderately severe, and severe depression. The PHQ-9 has been shown to have good psychometric properties [106].

*Overall Depression Severity and Impairment Scale (ODSIS)* [107]*.* It is a self-report measure with five-items that assess experiences related to depression, measuring its frequency and severity, as well as the level of avoidance behaviours, interference with work, school, and home, and associated social interference. The internal consistency of the scale has been shown to be excellent, with Cronbach alphas between 0.91 and 0.94 and good convergent and discriminant validity [108].

*Overall Anxiety Severity and Impairment Scale (OASIS)* [109]. It is a five-item questionnaire, with items rated from 0 to 4 that evaluate the frequency and severity of anxiety symptoms. The instrument also provides measures related to anxiety symptoms, such as avoidance, work, academy, social, and daily life disabilities. According to a psychometric analysis, it has good internal consistency (α = .80), test-test reliability (k = .82), and convergent validity. Online adaptation to a Spanish sample shows good internal consistency (α = 0.86), adequate convergent and discriminant validity, and a cut-off score of 7.5 [108, 110].

*The Generalized Anxiety Disorder Questionnaire (GAD-7)* [111] It is a seven-item dimensional self-administration scale designed to assess the presence of the symptoms of Generalized Anxiety Disorder (GAD) according to the DSM-IV. It is a one-dimensional self-administered scale, and although it does not provide a definitive diagnosis of GAD, it is an efficient, quick to apply, reliable, and valid instrument for detecting symptoms of an anxiety disorder. The scoring scale ranges from 0 to 3 (0 = nothing, 3 = almost every day), adding up to 0 to 21 points. There are four severity cut-off points (minimum = 0 to 4, mild = 5 to 9, moderate = 10 to 14, serious = 14 to 20) representing minimum to severe general anxiety thresholds. The GAD-7 has demonstrated good internal consistency and test-retest reliability, as well as convergent, construction, criterion, procedural, and factorial validity for GAD diagnosis [111–113].

*The Perceived Stress Scale - 4 items (PSS-4)* [114] It is a four-item self-report questionnaire that assesses the extent to which recent life situations are considered stressful. It uses a Likert scale from 1 to 5 (1 = never, 5 = very often). The PSS-4 is a short version that has been used for telephone interviews or study conditions requiring short versions [115]. It has demonstrated good internal consistency reliability in different studies [115, 116].

#### Self-compassion

*Self-compassion Scale - Short Form (SCS-SF)* [117] is designed to assess general self-compassion (total score) and three facets of this construct: common humanity (SCSCH), mindfulness (SCS-M), and self-kindness (SCS-SK). This version is shorter than the original version of the 26-item SCS [118]. It contains 6 subscales representing positive and negative aspects of each facet. A five-point Likert-type scale is used, ranging from 1 to 5 (1 = almost never, 5 = almost always). This short version (SCS-SF) has been shown to be valid and reliable [117].

#### Personality

*10-Item Big Five Inventory (BFI-10)* [119] was developed to provide a personality inventory for research environments with extreme time constraints. This questionnaire is an abridged version of the 44-item BFI [120]. It uses a five-point scale ranging from 1 to 5 (1 = strongly disagree, 5 = strongly agree). Previous studies have shown that this version has psychometric properties that are comparable in size and structure to those of the full BFI scale [119].

#### Program evaluation

*The Credibility and Expectancy Questionnaire (CEQ)* [121] evaluates factors of patient expectations and credibility of the treatment. This self-report consists of six-items with responses rated on a 10-point scale and on a scale of 1–100%. These factors have been shown to be stable in different populations, with high internal consistency within each factor [121].

*Client Satisfaction Questionnaire (CSQ)* [122, 123] consists of eight-items measured on a 4-point scale, with total scores ranging from 8 to 32 measuring the overall level of patient satisfaction with the treatment. Studies show good internal consistency reliability [122–125].

*Working Alliance Inventory for Technology Based Interventions (WAI-TECH)* [126] is a questionnaire that evaluates the therapeutic alliance between the technological tool and the patient. It covers two dimensions of the working alliance: (1) therapeutic objectives and (2) tasks. It consists of 8 items rated on a 5-point Likert scale ranging from 1 to 5 (1 = never, 5 = always). This questionnaire maintains adequate reliability and validity [126].

#### Other measures

Measurements of sociodemographic variables are included: age, sex, household size and income, marital status, employment status, total population of the place of residence, nationality, level of education, and living situation. In addition, health-related variables will be measured: presence of psychological disorders (past and present) and whether treatment is currently being carried out.

*Dropouts*, the participants will be asked about their reasons for cancelling the intervention.

### Support

Each country will provide their supporters, which will have at least a Bachelor’s degree in Psychology. All supporters will received a training in the protocol via an online platform coordinated by the Spanish team. The training consist in 2 days session and last 2 h each. On the first session, information about the overall goal of the study, conditions and time schedule will be explain. The documentation with the procedure explained by steps will be share and doubts on how to proceed will be solve. Second day will be dedicated on the use of the platform, supporters will be requested to create accounts, assign interventions, among other task they must know how to do given will be required for the trial. All supporters will in addition had a contact information to solve any doubt or problem they can found either in the trial or with the use of the platform.

### Statistical analysis

Intention-to-treat analysis and analysis per protocol will be carried out according to CONSORT recommendations [127]. The three groups at baseline will be compared to verify that there are no significant differences between them and, therefore, to confirm that they are comparable after randomization. One-way ANOVAs will be used for continuous variables and Chi-square tests of independence for categorical variables. For continuous outcome measures in the post-test, the assumption of homoscedasticity will be evaluated with the Levene test. If this assumption is met, the usual F-test will be applied for all three groups to compare post-test means. When the assumption of homoscedasticity is not met, Brown-Forsythe F test will be applied. Statistically significant F-tests will be followed by post hoc comparisons. In addition, Tukey procedure, will be applied when the assumption of homoscedasticity is met, and the Games-Howell procedure if it is not.

The intention-to-treat principle will be used to analyse primary and secondary post-treatment outcomes and at 3-month follow-up, using mixed-effect models with a maximum likelihood estimate of complete information, to properly evaluate data missing from repeated ANOVA measurements [128]. The results of the ANOVA and post-hoc comparisons were supplemented by calculating effect sizes using Cohen’s standardized mean difference [90]. The effect sizes will be calculated to compare both changes within a group and between groups, all based on a combined standard deviation. Per protocol analysis (completers) will be carried out to help draw conclusions about maximum treatment efficacy [129].

When the trial is completed, the analytical methodology of the RCT will be reviewed to select the most appropriate analytical procedures prior to data analysis.

## Discussion

This paper describes an Internet-based protocol designed to promote resilience and coping skills among college students in Spanish-speaking countries (Spain, Argentina, Colombia, and Mexico). The content is based primarily on Carol Ryff’s model of psychological well-being [86, 92, 130, 131]. Our goal is to evaluate the effectiveness, understood as acceptance of the program in particular and of Internet-based interventions in general, compared to an active control condition (HPL) and a waiting list (WL), using five universities -armed system.

There is a high prevalence of emotional disorders among young people around the world [132], and therefore it is essential to create preventive interventions in these populations at risk of developing a mental disorder [74, 133–135] such as the college population [10, 11]. This evidence suggests shifting attention to vulnerable populations and generating intervention strategies that include intervention planning, evaluation, and even effective health policies [136], reducing the costs of disability globally, creating strategic plans for improving quality of life, and building supportive environments and resilient communities [137].

Hence, the college is a key environment for the early detection and prevention of mental illnesses that may have high future economic costs [138]. Promoting resilience is an approach that supports this goal and well-being in this stage of life [139]. Given the importance of developing preventive interventions that promote resilience in at-risk populations, such as college students, it is important to propose interventions that are affordable and accessible to all. The Internet can be an effective means of delivering mental health interventions. This alternative format to traditional face-to-face psychological programs will increase the accessibility of psychological interventions for college students and will increase the tools of professionals to reach a greater number of people (following the recommendations of the National Institute of Mental Health Psychosocial Intervention Development Workgroup and the New Freedom Commission on Mental Health) [140, 141]. College students are immersed in digital worlds, and so Internet-based interventions can be potentially advantageous compared to face-to-face interventions [55]. Even though in Europe interventions for the prevention of depression and the promotion of resilience have already been established [142, 143], there are no studies that show results in college students of implementing technological tools such as the Internet for delivering psychological interventions.

Consequently, the strengths of this study focus on two aspects: First, to our knowledge, this would be the first RCT to evaluate an online intervention to improve the resilience of Spanish-speaking college students (in Spain and Latin America). Second, CORE is a three-armed, single blind multi-country (Spain, Argentina, Colombia, and Mexico) randomized controlled trial (RCT) that will make it possible to study the generalization of the results in different Spanish-speaking students. Third, we are comparing the results with an active control, a rigorous Healthy Lifestyle program developed in Spain and characterized by the promotion of habits of physical health, diet, and sleep, that although outcomes analyses are in progress, preliminary data show acceptance and effectiveness of this low-intensity internet program to reduce the symptoms of depression [93]. However, this study has some limitations: first, dropout rates are expected to be high, as reported in previous research [144, 145]. For this reason, dropout rates have been taken into account in the calculation of the sample size. Second, Internet-based interventions for mental health also raise some ethical issues with regard to the exchange of data between Europe and America. For this reason, international guidelines will be followed to address these concerns [146, 147], as well as all aspects of Personal Data Protection under existing European legislation in compliance with General Data Protection Regulation 2016/679.

We consider that this study will provide future lines of research that will strengthen the implementation of these interventions. Little is known about the potential for cultural adaptation and the efficacy of interventions for mental disorders in low- and middle-income countries [148]. According to World Bank data, Colombia and Mexico are considered upper-middle-income countries and Argentina is a high-income country. However, the WHO includes them in America region, where the prevalence of mental disorders is higher than in the European region, but this variation may be due to the fact that many people have both conditions (comorbidity) and to the relatively larger populations between these two regions [5]. These data suggest the importance of prioritizing the prevention and treatment of mental disorders globally in both developing and developed regions [6].

An important aspect is that CORE and HLP have been developed in Europe and adapted to be implemented in the Latin-American countries. Language is an advantage that can favour accessibility to Spanish-speaking countries, and empirical evidence indicates that the advantages of interventions administered through the Internet can facilitate access to this population to promote mental health worldwide. Data from a systematic review indicate that, although there are few studies examining the effect of online interventions in low-income countries, the effectiveness of these online interventions in high-income countries and the current increase in Internet access should be taken into account. Moreover, these interventions may help to reduce the “mental health gap” in providing low-cost, more widely distributed mental health care [149]. In addition, CORE includes intervention strategies that have been found to be useful for improving resilience, and HLP includes psycho-educational components to prevent depressive symptoms. Therefore, both programs are expected to show more effectiveness than the Waiting List control group in Spanish-speaking countries.

In addition, this study will provide information regarding the possibilities of implement interventions designed in developed countries, in other countries or situations. The study will provide information about participant’s acceptability and satisfaction with the intervention,

Finally, this protocol aims to increase access to and acceptance of Internet-based interventions, overcoming current mental health barriers in high-, middle-, and low-income countries, in order to implement and establish a comprehensive model of mental health promotion in Spanish-speaking countries in Europe and America.

### Trial status

Recruitment for this study began in June 2019. It is currently in progress.

## Data Availability

Data will be available once the trial is finished under request.

## Abbreviations

CORE: Unguided internet-based resilience intervention
HLP: Healthy lifestyle program
WL: Waiting list control
CMHD: Common Mental Health Disorders
